# Identification and Verification of Key Genes in Colorectal Cancer Liver Metastases Through Analysis of Single-Cell Sequencing Data and TCGA Data

**DOI:** 10.1245/s10434-024-16194-9

**Published:** 2024-10-09

**Authors:** Hui Zhang, Changhua Zhuo, Ruirong Lin, Fayong Ke, Ming Wang, Chunkang Yang

**Affiliations:** 1 Department of Hepatopancreatobiliary Surgical Oncology, Fujian Cancer Hospital, College of Clinical Medicine for Oncology, Fujian Medical University, Fuzhou, Fujian People’s Republic of China; 2 Department of Gastrointestinal Surgical Oncology, Fujian Cancer Hospital, College of Clinical Medicine for Oncology, Fujian Medical University, Fuzhou, Fujian People’s Republic of China

**Keywords:** Colorectal cancer liver metastases metastases, Tumor microenvironment, Single-cell RNA-sequencing, Colorectal cancer stem cells

## Abstract

**Background:**

Colorectal cancer (CRC) is highly prevalent worldwide, with more patients experiencing colorectal cancer liver metastases (CRLM). This study aimed to identify key genes in CRLM through single-cell sequencing data reanalysis and experimental validation.

**Methods:**

The study analyzed single-cell RNA-sequencing (scRNA-seq) data from the Gene Expression Omnibus (GEO) database. Gene ontology (GO) and the Kyoto Encyclopedia of Genes and Genomes (KEGG) were used for gene functional enrichment analysis. The Cancer Genome Atlas (TCGA) data enabled bulk-RNA expression and survival prognosis analysis. Real-time polymerase chain reaction (qPCR) detected mRNA expression, whereas Western blot determined protein levels. Cell function experiments assessed SPARC’s impact on CRC cell behavior.

**Results:**

Cluster analysis showed 23 classes among 17 CRLM samples, representing six cell types. A GO and KEGG analysis identified interleukin-1 beta (IL1B), CD2 molecule (CD2), and C-X-C motif chemokine ligand 8 (CXCL8) as significant prognostic factors in CRC. Secreted protein acidic and cysteine rich (SPARC) was one of the top differentially expressed genes (DEGs) in tissue stem cells, confirmed in primary and metastatic lesions. Metastatic lesions showed higher expression of SPARC and CRC stem cell marker leucine-rich repeat containing G protein-coupled receptor 5 (LGR5), which was significantly correlated positively with LGR5 expression. Knockdown of SPARC reduced CRC cell sphere- and colony-formation, invasion, and migration abilities. Overexpression of SPARC significantly increased the malignancy of CRC cells.

**Conclusions:**

Several key genes were identified in the process of CRLM. In CRLM samples and those corresponding to CRC stem cells, SPARC was significantly upregulated. In the therapy of CRLM, SPARC might be a potential target.

**Supplementary Information:**

The online version contains supplementary material available at 10.1245/s10434-024-16194-9.

Colorectal cancer (CRC), also known as colon cancer or rectal cancer, is one of the most common malignant tumors. Advances in surgical techniques and neoadjuvant chemotherapy have significantly improved the overall survival rate of CRC.^1^ However, improving survival for patients with recurrent metastatic disease remains a challenge.

The liver is a common site of metastasis in colorectal cancer.^2^ Liver metastases (LM) occur in 20–25% of cases comprising newly diagnosed CRC, and eventually in 40–50% of cases involving surgical removal of CRC.^3,4^ Liver metastases also are the main cause of death from CRC.^5^ How to improve the efficacy of patients with LM has become one of the focuses in the field of CRC research.

According to latest research, 50–60% of patients with CRC will eventually experience LM, and the proportion of LM in patients with postoperative recurrence is as high as 70%.^6^ In the autopsy of patients who died of CRC, about 70% were found to have LM.^7^ It is generally believed that the incidence of simultaneous LM is about 15–25%, and that the incidence of heterochronous LM is about 20–25%.^6^

Immune checkpoint blockade is regarded as a promising therapy for numerous solid tumors, including melanoma and non-small cell lung cancer.^8,9^ Immune checkpoint inhibitors that target PD-1 or PD-L1 also have been tested in CRC.^8^ However, only a limited number of patients have demonstrated a response to anti-PD-1 or anti-PD-L1 immunotherapy in recent clinical trials.^8^

In CRC, cancer cells interact with both immune cells and stromal cells to form an immunosuppressive tumor microenvironment (TME), thus enhancing cancer cell immune evasion. Intertumoral heterogeneity also is an important feature of CRC, leading to treatment resistance and divergent therapeutic outcomes among patients.^10^ Understanding cancer cell heterogeneity as well as the dynamic tumor immune microenvironment could provide new therapeutic targets for the treatment of CRC.

The emergence of single-cell RNA-sequencing (scRNA-seq) technology has fundamentally changed the field of tumor biology, providing a strategy to demonstrate TME heterogeneity as well as intercellular communication at the single cell level.^11^ The immunoregulatory characteristics of myeloid cells might account for most of the tumor-infiltrating immune cells in CRC. Myeloid cells, including macrophages, dendritic cells (DCs), and monocytes, play a vital role in tumor immune surveillance through phagocytosis and antigen processing and presentation.^12^ Myeloid cells also may play a critical role in regulating CRC metastasis.^4^ Given that these myeloid cells can be polarized toward a protumor/antitumor response, we hypothesized that regulating myeloid cells in the TME can be a promising immunotherapy strategy for inhibition of CRC LM.

To identify the profile of tumor-infiltrating myeloid cells and the immune heterogeneity of CRC and LM, we analyzed published scRNA-seq datasets. In this study, the differences between CRC and LM were analyzed to explain the mechanisms of metastasis to LM. Through systematic analyses, our study helps to elucidate the biology of the TME in CRC and contributes to the development of immunotherapy in clinical applications. Therefore, this study analyzed the cell clusters and genes of colorectal cancer liver metastases (CRLM) from the single-cell level, providing certain support for the study of CRLM.

## Methods

### Data Processing and Analysis

Data were sourced from the Gene Expression Omnibus (GEO) and The Cancer Genome Atlas (TCGA) databases. The study obtained scRNA-seq data from Gene Expression Omnibus (GEO) database GSE164522.^12,13^ The dataset contains 17 single cell-sequencing samples of CRLM. The gene expression matrix of the corresponding samples was downloaded, and the expression matrix was divided into primary CRC matrix and LM matrix. Seurat analysis was used for subsequent analysis. Highly variable genes were used for identification after normalization of the data. The normalized data were used for dimensionality reduction (TSNE),^14^ and the available dimensions of the cells were identified to cluster the cells and annotate the cell type.

Subsequently, downstream data analysis was performed. The results of the cluster were preliminarily analyzed as a whole. Then the cluster results were annotated by cells, mainly using tools for SingleR^15^ and the customer gene list for manual annotation. Gene ontology (GO)^16^ and Kyoto Encyclopedia of Genes and Genomes (KEGG)^17^ analyses were performed for T and B cell types, and re-clustering was performed.

Next, GO and KEGG analyses were performed on CRC type genes, and string interaction analysis was performed on differential genes to find the top 10 genes. The Cancer Genome Atlas (TCGA) data were used for bulk-RNA expression analysis and survival prognosis analysis.

### Tissue Samples

The study collected 36 CRC specimens from patients who underwent surgical treatment between January 2022 and February 2023 at Fujian Cancer Hospital. For primary tumor sampling (*n* = 18), specimens were obtained from the central region of the tumor to avoid areas of necrosis and hemorrhage. For patients with CRLM (*n* = 18), samples were taken from metastatic lesions in liver tissue, which are representative of metastatic pathology. The patients’ clinical information, including age, gender, tumor stage, histopathologic grade, and treatment history, is detailed in Table S1. The study was conducted in accordance with the Declaration of Helsinki, and the protocol was approved by the Ethics Committees of Fujian Cancer Hospital (approval no. SQ2020-086-01). Informed consent was obtained from all the individual participants included in the study.

### Cell Culture

Human colon cancer cell lines (LoVo, RKO, SW48, HCT116, SW480, and SW620) and the human immortalized normal colon epithelial cell line (HIEC) were purchased from the cell bank of the Type Culture Collection of the Chinese Academy of Sciences (Shanghai, China). All the cell lines were maintained in RPMI-1640 medium (Gibco, Waltham, MA, USA) containing 10% fetal bovine serum (FBS, Gibco), 100 U/ml of penicillin (Invitrogen, Carlsbad, CA, USA), and 100 mg/ml of streptomycin (Invitrogen) in a humidified incubator with 5% carbon dioxide (CO_2_) at 37 °C.

### Cell Transfection

The cells (LoVo and HCT116) for transfection were cultured in appropriate growth medium until they reached 70% to 80% confluency for optimal transfection efficiency. Two SPARC small interfering RNAs (siRNAs) and corresponding si-NC (Table 1**)** were secured from the GeneChem Company (Shanghai, China). The growth medium was removed from the cells and washed once with serum-free medium or phosphate-buffered saline (PBS). Transfection complexes were added dropwise or gently mixed into the cells. Even distribution of the transfection mixture was ensured. The transfection complexes were incubated with the cells in a CO_2_ incubator at 37 °C for a period specified by the manufacturer of the transfection reagent. At the end of incubation, the transfection medium was replaced with fresh growth medium containing serum. The cells were incubated under normal culture conditions for the desired time. To measure gene expression levels, real-time polymerase chain reaction (qPCR) was performed, and Western blotting was performed to detect protein expression to verify the success of transfection.

**Table 1:** The two SPARC siRNAs and corresponding si-NC

siRNAs	Start Sequence(DNA)	Sequence
siRNA-1	208	5′-CCAGGUGGAAGUAGGAGAAUUUGAU-3′
Si-NC	–	5′-CCAGGUGAAGAUGAGUAAUUGGGAU-3′
siRNA-2	416	5′-ACCUUCGACUCUUCCUGCCACUUCU-3′
Si-NC	–	5′-ACCAGCCUCCUUGUCACCUCUUUCU-3′

siRNA, small interfering RNA

### Quantitative PCR

Total RNA was isolated from cells using the RNeasy Mini Kit (QIAGEN, Hilden, Germany), and qPCR was performed using Superscript IV Reverse Transcriptase (ThermoFisher, Waltham, MA, USA). Gene-fold changes were determined by the 2^−ΔΔCt^ algorithm, and β-actin was used as reference genes to normalize 2^−ΔΔCt^-based assessments. The specific primer sequences for COL3A, IGFBP7, SPARC, COL1A2, CALD1, and β-actin are shown in Table 2.

**Table 2:** The specific sequence of different primers

COL3A	Sense	5′-GCCAAATATGTGTCTGTGACTCA-3′
	Anti-sense	5′-GGGCGAGTAGGAGCAGTTG-3′
IGFBP7	Sense	5′-ATCCCGACACCTGTCCTCAT-3′
	Anti-sense	5′-CCCAGCCAGTTACTTCATGCT -3′
SPARC	Sense	5′-TGAGGTATCTGTGGGAGCTAATC-3′
	Anti-sense	5′-CCTTGCCGTGTTTGCAGTG-3′
COL1A2	Sense	5′-GGCCCTCAAGGTTTCCAAGG-3′
	Anti-sense	5′-CACCCTGTGGTCCAACAACTC-3′
CALD1	SENSE	5′-TGGAGGTGAATGCCCAGAAC-3′
	Anti-sense	5′-GAAGGCGTTTTTGGCGTCTTT-3′
β-actin	Sense	5′-TGACGTGGACATCCGCAAAG-3′
	Anti-sense	5′-CTGGAAGGTGGACAGCGAGG-3′

### Western Blot

The study used RIPA lysis buffer (Beyotime Biotechnology, Shanghai, China) for protein extraction from colon tissue. To separate protein samples (60 μg), SDS-PAGE was used, and for transfer, PVDF membranes (microwells) were used. Extracted protein then was incubated on the membrane overnight at 4 °C after blocking using the primary antibody (LGR5 antibody, 1:1000, cat. no. ab75850; SPARC antibody, 1:1000, cat. no. ab207743; CALD1 antibody, 1:1000, cat. no. ab265026; β-cantenin antibody 1:1000, cat. no. ab114594; GAPDH antibody, 1:1000, cat. no. ab8245; β-actin antibody, 1:1000, cat. no. ab8226; Abcam, Cambridge, MA, USA). The next day, horseradish peroxidase (HRP)-conjugated secondary antibodies were used for the incubation. Visualization was mainly achieved by a fluorescence imager (Alpha), and the expression levels of specific proteins were normalized to β-actin or GAPDH levels.

### Spheroid Assay

Primary cells were isolated from tumor tissue and maintained in StemPro hESC SFM (Thermo Fisher Scientific, Waltham, MA, USA) supplemented with 8 ng/mL of basic fibroblast growth factor (Sigma-Aldrich, Allentown, PA, USA) in ultra-low attachment six-well plates (Corning Inc., Corning, NY, USA). Cells were seeded into ultra-low-attachment six-well plates (Corning Inc.) at a density of 1 × 10^3^ cells per well. After 10–15 days of culture, the cells were observed under a phase-contrast microscope (Leica Microsystems, Wetzlar, Germany).

### Colony-Formation Assay

The LoVo and HCT116 cells and their sorted cells were seeded onto 96-well plates at limited dilution. After 14 days, the number of colonies was counted under an inverted phase-contrast microscope.

### Transwell Invasion Assay

The LoVo and HCT116 cells were seeded onto 24-well plates (5 × 10^4^ cells/well) and incubated for 20 h in a cell incubator. The culture was continued for 24 h after transfection. Then, 1 × 10^5^ cells were seeded in Matrigel (1:8, 80 μL)-coated Transwell chambers and 100 μL serum-free RPMI-1640 medium, respectively. Complete medium was added to the lower chamber of the Transwell chamber, and after 24 h of incubation, the cells in the upper chamber were wiped with a cotton swab. The cells were fixed with 4% paraformaldehyde for 15 min and stained with crystal violet for 10 min. Five fields were randomly selected under the microscope (Leica Microsystems), photographed, and counted. The experiment was repeated three times, and the mean value was calculated.

### Wound-Healing Assay

The LoVo and HCT116 cells were seeded in six-well plates (3 × 10^5^ cells/well) and grown for 20 h in a cell incubator. A uniform horizontal line was drawn on the back of a six-well plate. The cells were scratched with a 10-μL pipette along a ruler perpendicular to the horizontal line on the back, and the scratched cells were removed by PBS rinse. Culture medium was added after transfection treatment. Samples were collected at 0 and 48 h, and the scratch-healing area was calculated as follows: mobility = scratch-healing area/scratch initial area ×100%. The experiment was repeated three times, and the mean value was calculated.

### Statistical Analysis

All statistical analyses and visualizations were performed using Microsoft Excel, GraphPad Prism 8.0, or R software, version 4.1.0, unless otherwise stated. Clinical characteristics were expressed as mean ± standard deviation or a *n* (%). The Benjamini-Hochberg method was used to control the false discovery rate (FDR). Adjusted *p* values lower than 0.05 were considered to indicate significance.

## Results

### Holistic Cluster Analysis

**Fig. 1:**
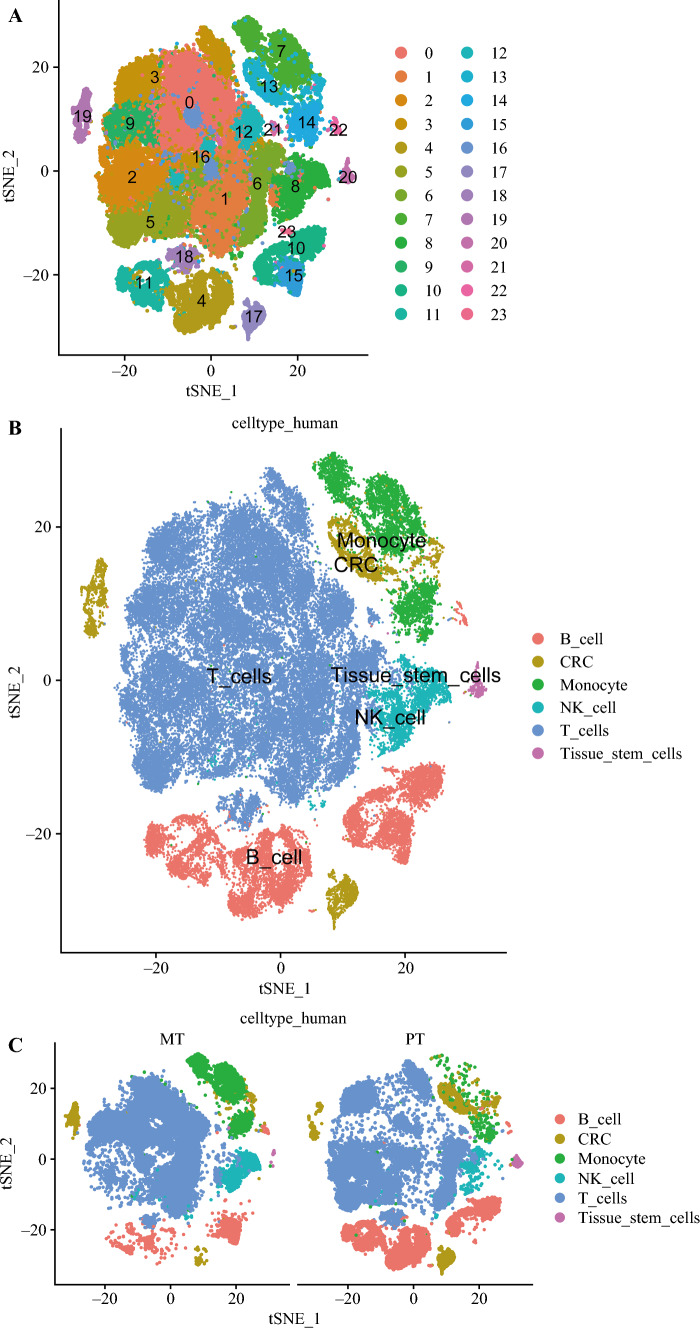
Analysis of single-cell sequencing data of 17 colorectal cancer patients with liver metastasis tissues. **A** Cells clustered into 23 types via the tSNE dimensionality reduction algorithm, each color representing the annotated phenotype of each cluster. **B** The 23 cell types identified into six cell types after annotation. **C** Distribution of individual cell types in colorectal cancer and liver metastasis samples. MT, metastasis to liver; PT, primary tumor

The scRNA-seq data from GEO database GSE164522 contained gene expression matrices for 17 CRLM samples. Cluster analysis of the normalized single-cell sequencing data was performed, and the results showed that 17 samples of CRLM were clustered into 23 classes (Fig. 1A). The expression bubble plots of the top four genes in different clusters are shown in the Fig. S1. The SingleR and HumanPrimaryCellAtlasData databases were used for cell type annotation at the same time, and CustomGene.txt was used for further manual annotation (Fig. 1B).

To further display the cell annotation results in different pathogenic tissues, the cell annotation results were separated. The distribution of various types of cells in colorectal cancer and LM samples are shown in Fig. 1C. In the two different sample types, except for monocyte type cells that accounted for a higher proportion in LM samples than in colorectal cancer samples, the distribution of other cell types was roughly the same. Therefore, we speculated that monocyte type cells may play a role in CRLM.

### Re-Cluster Analysis of T Cells

**Fig. 2:**
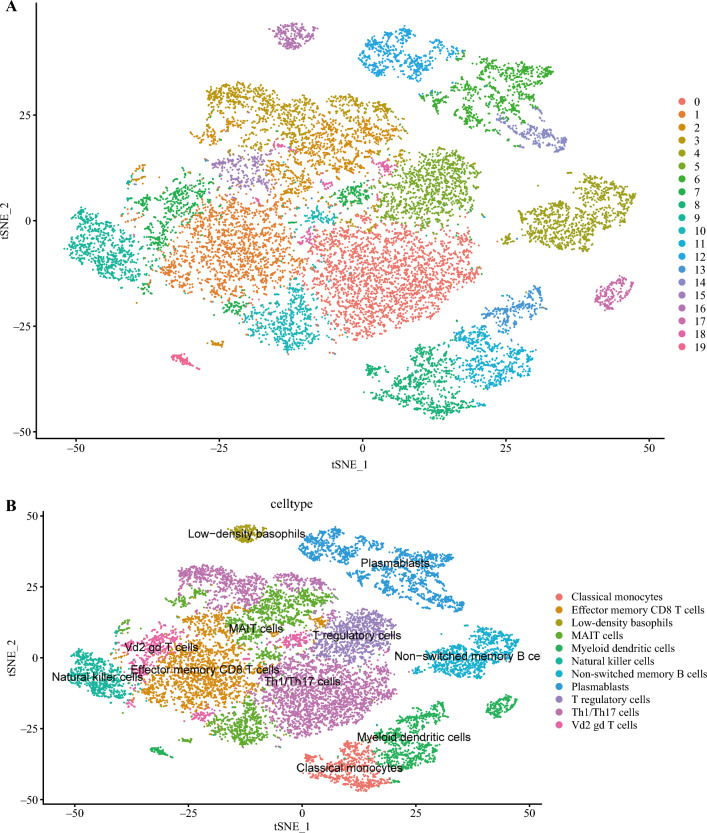

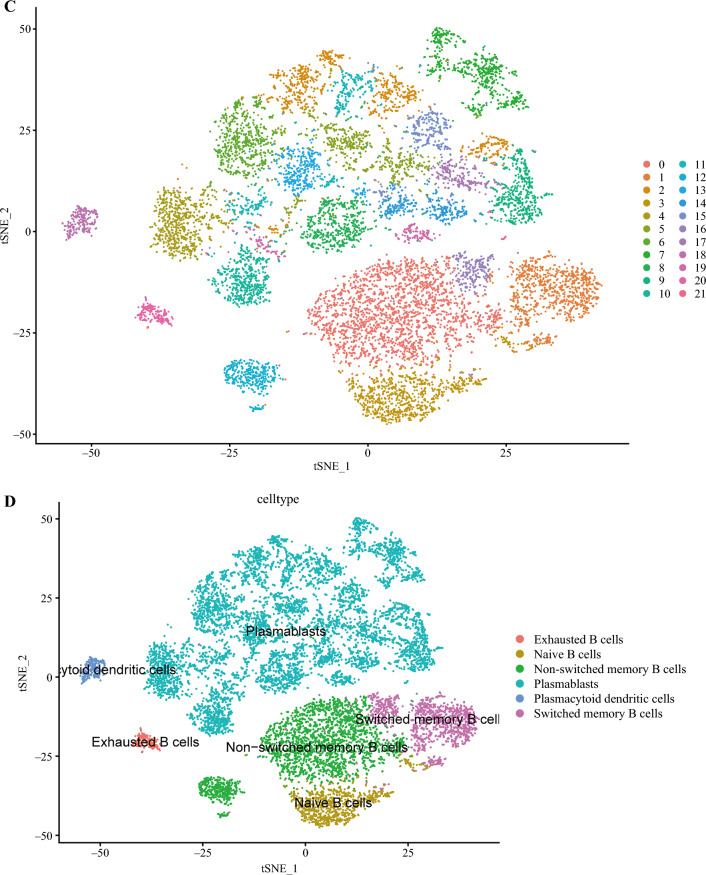
Re-cluster analysis of T cells and B cells. **A, B** Re-cluster analysis and annotation of T cells. **C,D** Re-cluster analysis and annotation of B cells.

To further investigate the subtypes of T cells and their associated biologic processes and functions, the gene data of T cells were re-clustered and annotated using the SingleR tool with the MonacoImmuneData annotation database. Figure 2A shows the results of the re-clustering, wherein T cells were classified into 19 clusters. Figure 2B displays the cell annotation results, with T cells reannotated into 11 cell types such as TH1/Th17 cells and CD8 T cells.

To explore the main biologic processes and functions involved in T cell subtypes, functional enrichment analysis was performed on different clusters. Figure S2A presents a KEGG enrichment bubble plot, which shows that these cells were mainly involved in processes such as cytokine-cytokine receptor interaction and NF-kappa B signaling pathway. Figure S2B displays a GO-BP enrichment circle diagram, indicating that these cells are involved mainly in pathways including lymphocyte-mediated immunity and adaptive immune response based on somatic recombination of immune receptors built from immunoglobulin superfamily domains.

### Re-Cluster Analysis of B Cells

To further explore the subtypes of B cells and their associated biologic processes and functions, the gene data of B cells were re-clustered and annotated using the SingleR tool with the MonacoImmuneData annotation database. Figure 2C shows the results of the re-clustering, wherein B cells were classified into 21 clusters. Figure 2D displays the cell annotation results, in which B cells were reannotated into six cell types such as exhausted B cells and naive B cells.

To investigate the main biologic processes and functions involved in B cell subtypes, functional enrichment analysis of different clusters was performed. Figure S3A presents a KEGG enrichment bubble plot, which indicates that these cells are involved mainly in pathways such as hematopoietic cell lineage and inflammatory bowel disease. Figure S3B displays a GO-BP enrichment circle diagram, showing that these cells are primarily involved in pathways including humoral immune response mediated by circulating immunoglobulin and B cell-mediated immunity.

### Functional Analysis of CRC Cell Types

**Fig. 3:**
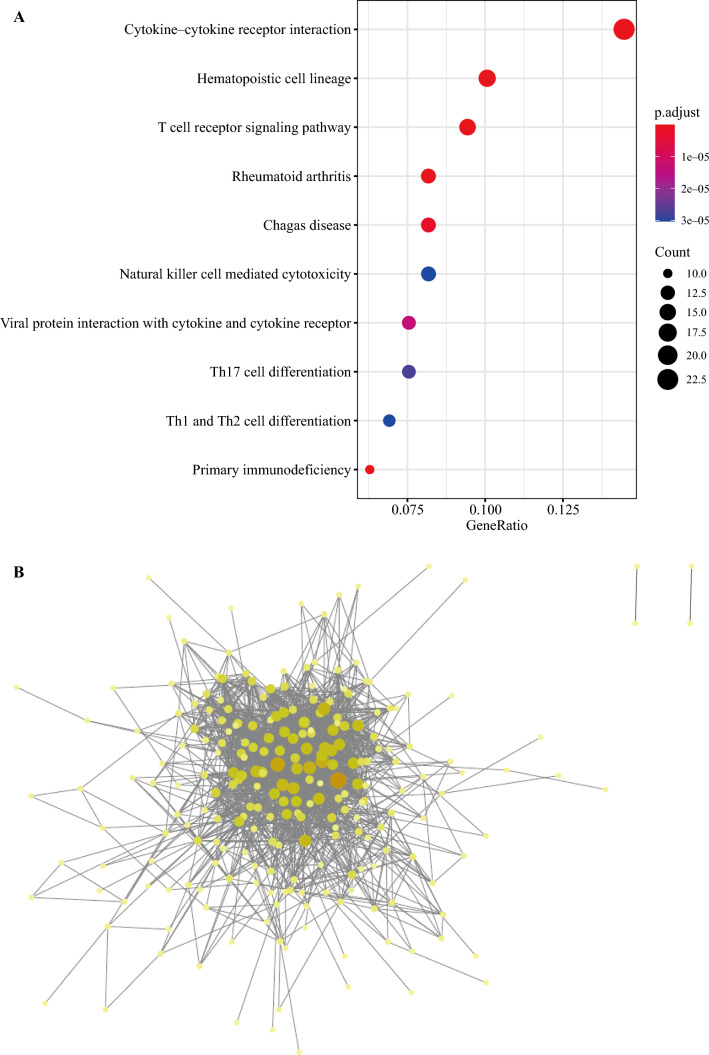
Functional enrichment analysis and PPI analysis of DEGs in CRC cells. **A** Functional enrichment analysis of DEGs in CRC cells. **B** PPI analysis of DEGs in CRC cells. PPI, protein-protein interaction; DEGs, differentially expressed genes; CRC, colorectal cancer

Violin plots were used to depicted the top five differentially expressed genes in different cell types including T cells (Fig. S4A), B cells (Fig. S4B), monocytes (Fig. S4C), NK cells (Fig. S5A), CRC (Fig. S5B), and tissue stem cells (Fig. S5C). These plots show that the majority of differentially expressed genes exhibit higher and more concentrated expression levels in their corresponding cell types. Functional enrichment analysis of differentially expressed genes in CRC cells was performed. The KEGG enrichment results are shown in Fig. S6, indicating that these genes were primarily involved in pathways such as cytokine-cytokine receptor interaction and natural killer cell-mediated cytotoxicity. Figure 3A displays the CRC cell GO enrichment bar plot, highlighting processes such as positive regulation of leukocyte activation and the external side of the plasma membrane.

**Fig. 4:**
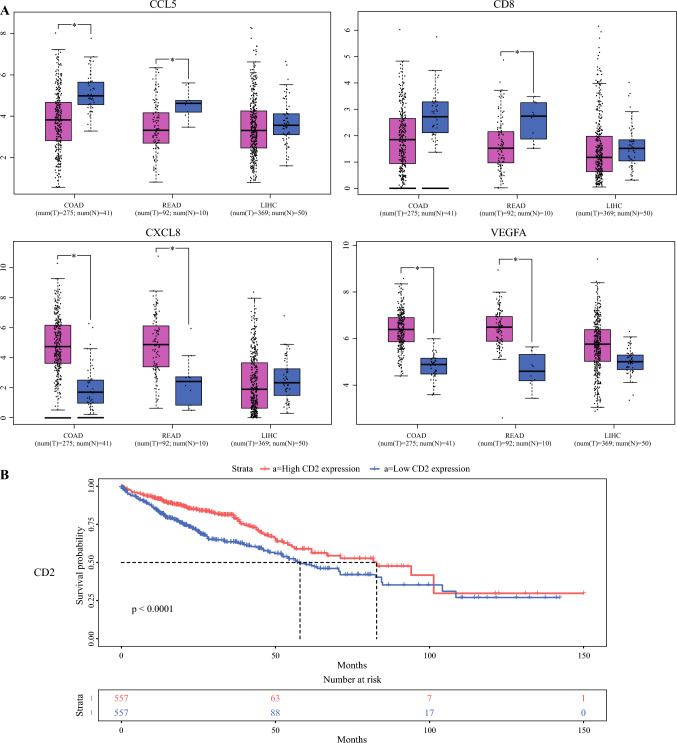

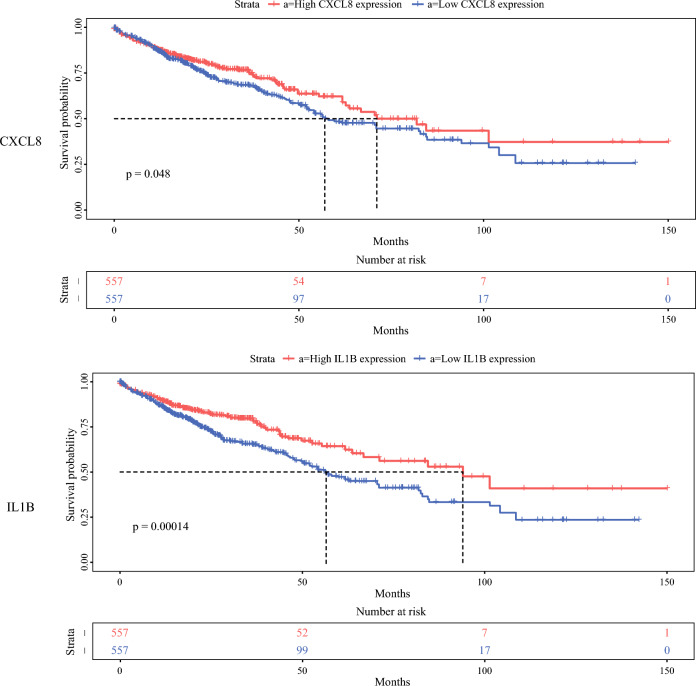
Expression verification and survival analysis of identified DEGs in TCGA. **A** Expression of CCL5, CD8, CXCL8, and VEGFA in TCGA (COAD, READ, and LIHC). **B** Survival analysis of CCL5, CD8, CXCL8, and VEGFA in TCGA (COAD, READ, and LIHC). DEGs, differentially expressed genes; TCGA, The Cancer Genome Atlas; COAD, colon adenocarcinoma. READ, rectal adenocarcinoma. LIHC, liver hepatocellular carcinoma

To further investigate the interactions among these differentially expressed genes, a protein-protein interaction (PPI) network was constructed using the STRING database, as shown in Fig. 3B. This network illustrated complex regulatory relationships among these genes. Among the top 10 genes ranked by degree in the network, differential expression analysis and survival prognosis analysis were performed using TCGA data. Genes such as CCL5, CD8, CXCL8, and VEGFA exhibited significant differences in COAD, READ, and LIHC, as demonstrated in Fig. 4A. Additionally, IL1B, CD2, and CXCL8 showed significant impact on the survival prognosis of colorectal cancer (Fig. 4B).

### Expression Verification of Key Genes in Primary and Metastatic CRC

**Fig. 5:**
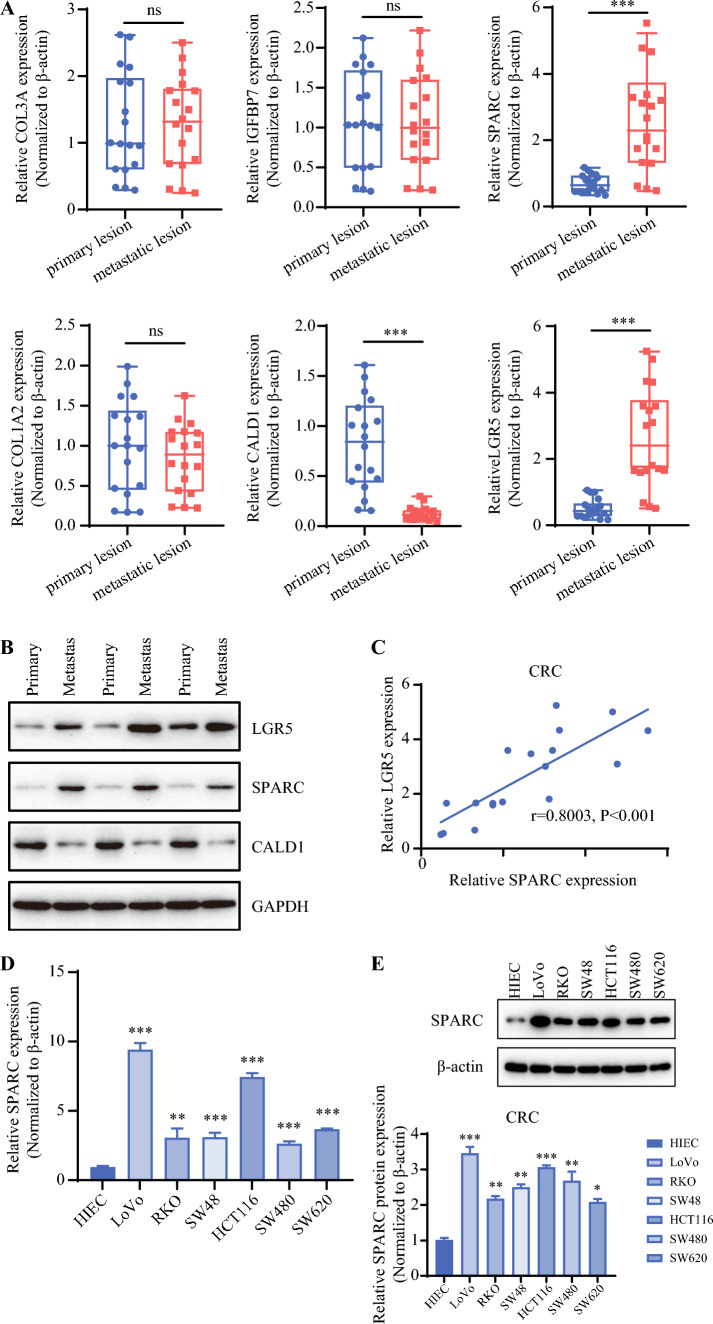
Expression of colorectal cancer stem cell genes in primary or metastatic lesions. **A** Expression levels of COL3A, IGFBP7, SPARC, COL1A2, CALD1, and LGR5 were assessed using qPCR in the primary tumors (*n* = 18) and metastatic lesions (*n* = 18). **B** The protein expression levels of LGR5, SPARC, and CALD1 were assessed using WB in the primary tumors (*n* = 3) and metastatic lesions (*n* = 3). **C** The correlation between SPARC and LGR5 expression was analyzed according to the qPCR results. **D** The expression of SPARC was detected in six human colorectal cancer cell lines (LoVo, RKO, SW48, HCT116, SW480, and SW620) using qPCR. The human intestinal epithelial cell line HIEC was used as the control. **E** The expression of SPARC was detected in six human colorectal cancer cell lines (LoVo, RKO, SW48, HCT116, SW480, and SW620) using WB. The human intestinal epithelial cell line HIEC was used as the control. qPCR, real-time polymerase chain reaction; NS, no significance; WB, Western blot. ^*^p < 0.05; ^**^p < 0.01;^***^p < 0.001

According to the results of bioinformatics analysis, the top five differentially expressed genes in tissue stem cells and a colon cancer stem cell marker (LGR5) were further verified in primary (*n* = 18) and metastatic (*n* = 18) lesions. The results showed that the expression of COL3A, IGFBP7, and COL1A2 did not differ significantly between the primary and metastatic lesions. Compared with the primary lesions, SPARC and LGR5 were significantly higher and CALD1 was significantly lower in the metastatic lesions (Fig. 5A).

Furthermore, we further verified the protein expression of SPARC, LGR5, and CALD1 by Western blot, and the trend was consistent with the qPCR results (Fig. 5B). Based on the qPCR results, the expression correlation between SPARC and LGR5 was verified, and the results showed a significant positive correlation between SPARC and LGR5 expression (Fig. 5C). Subsequently, we verified SPARC expression in CRC cell lines LoVo, RKO, SW48, HCT116, SW480, and SW620. The qPCR results (Fig. 5D) and the WB results (Fig. 5E) showed that SPARC was more highly expressed in CRC cell lines than in normal intestinal cell line HIEC (human intestinal epithelial cell). Among them, SPARC had the most significant high expression in the LoVo and HCT116 cell lines, so these two cell lines were selected for subsequent studies.

### Knockdown/Overexpression of SPARC Affected the Cell Sphere-Formation Ability, Colony-Formation Ability, Invasion, and Migration Ability of CRC Cells

**Fig. 6:**
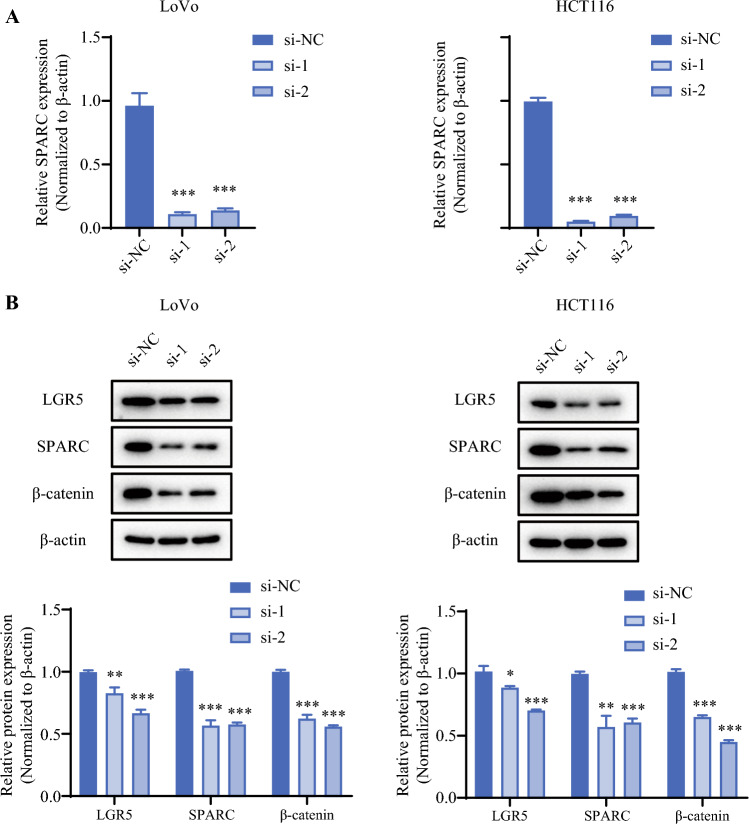
Validation of SPARC knockdown efficiency. **A** qPCR was performed to detect SPARC mRNA expression after knockdown by two siRNAs in the LoVo (*left*) and HCT116 (*right*) cell lines. **B** WB was performed to detect SPARC protein expression after knockdown by two siRNAs in the LoVo (*left*) and HCT116 (*right*) cell lines. siRNA, small interfering RNA; si-NC, siRNA-negative control; si-1, siRNA-SPARC-1; si-2, siRNA-SPARC-2; WB: qPCR, real-time polymerase chain reaction; siRNA, small interfering RNA; WB, Western blot. ^**^p < 0.01; ^***^p < 0.001

**Fig. 7:**
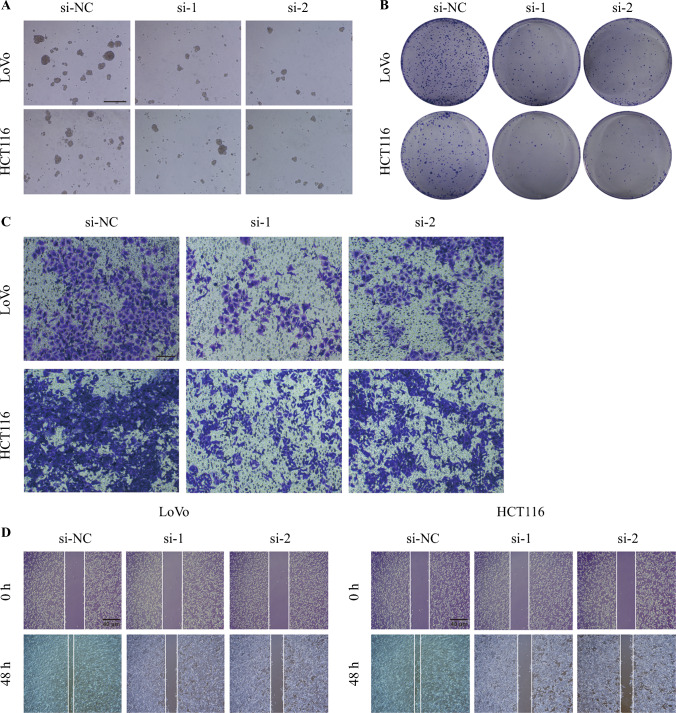
Knockdown of SPARC reduced the cell sphere-formation ability, colony-formation ability, invasion, and migration ability of CRC cells. **A** Knockdown of SPARC reduced the cell sphere-formation ability of the LoVo and HCT116 cell lines. Scale bar, 100 μm. **B** Knockdown of SPARC reduced the colony-formation ability of the LoVo and HCT116 cell lines. **C** Knockdown of SPARC reduced the invasion ability of the LoVo and HCT116 cell lines. Scale bar, 100 μm. **D** Knockdown of SPARC reduced the migration ability of the LoVo and HCT116 cell lines. Scale bar, 40 μm. CRC, colorectal cancer

**Fig. 8:**
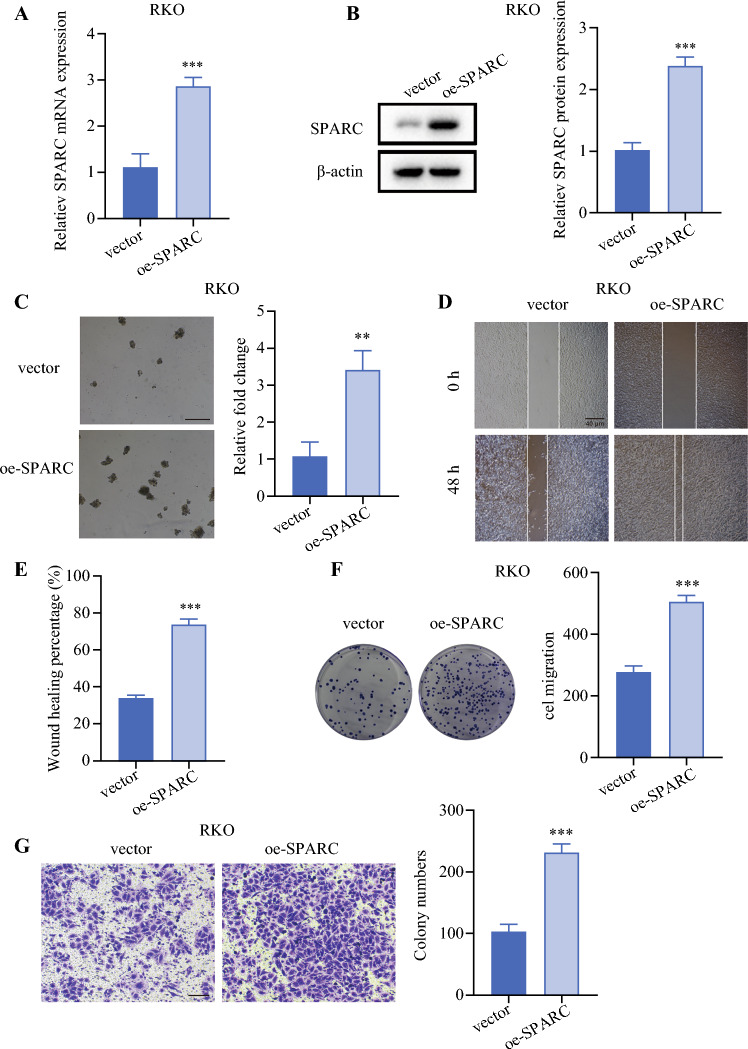
Overexpression of SPARC enhanced the cell sphere-formation ability, migration ability, colony-formation ability, and invasion of RKO cells. **A** qPCR was performed to detect SPARC mRNA expression after overexpression SPARC in RKO cells. **B** WB was performed to detect SPARC protein expression after overexpression of SPARC in RKO cells. **C** Overexpression of SPARC enhanced the cell sphere-formation ability of RKO cells. Scale bar, 100 μm. **D,E** Overexpression of SPARC enhanced the migration ability of RKO cells. Scale bar, 40 μm. **F** Overexpression of SPARC enhanced the colony-formation ability of RKO cells. **G** Overexpression of SPARC enhanced the invasion ability of RKO cells. Scale bar, 100 μm. oe-SPARC, overexpression of SPARC; WB, Western blot. ^**^p < 0.01; ^***^p < 0.001

To explore the effect of SPARC on cellular functions in CRC cell lines, SPARC knockdown experiments were performed in the LoVo and HCT116 cell lines, and SPARC overexpression experiments were performed in RKO cells. As shown in Fig. 6, both mRNA and protein expression of SPARC were significantly downregulated after knockdown. We then validated the effect of SPARC knockdown on the biologic behavior of CRC cell lines. The results showed that knockdown of SPARC reduced the cell sphere-formation ability (Fig. 7A), colony-formation ability (Fig. 7B), invasion (Fig. 7C), and migration ability (Fig. 7D) of CRC cells. Moreover, SPARC overexpression was performed in RKO cells. The mRNA (Fig. 8A) and protein (Fig. 8B) expressions of SPARC were visibly upregulated after overexpression. The results showed that the cell sphere-formation ability (Fig. 8C), migration ability (Fig. 8D and E), colony-formation ability (Fig. 8F), and invasion (Fig. 8G) were enhanced in the SPARC overexpression group.

### SPARC was Highly Expressed in Lymph Node Metastasis

To further realize the role of SPARC in lymph node metastasis, we analyzed TCGA-COADREAD downloaded from the Xena (https://xenabrowser.net/), GSE39582, and GSE29621 dataset. The results showed that SPARC had significantly higher expression in all of them than in the primary lesion (Fig. S7A-C). These results suggested that SPARC may play a role in lymph node metastasis.

## Discussion

Liver is a common site of CRC metastasis, and LM is the main cause of death from CRC. Liver metastasis will eventually develop in 50% to 60% of patients with colorectal cancer.^6^ Therefore, early detection of CRLM is particularly important. The detection of carcinoembryonic antigen (CEA) in bile and duodenal fluid may lead to the detection of earlier LM,^18^ and the discovery of new markers is expected to bring hope for the treatment of CRC. In this study, the cell types and gene changes of CRLM were analyzed by the single-cell technique, providing a basis for further research on the mechanism and treatment of CRLM.

Various cell types in the tumor microenvironment (TME) affect the progression and metastasis of cancer cells. The study identified TME-related indicators as important prognostic parameters. The use of scRNA-seq technology accelerates the identification of different cell populations and phenotypic states within tumors, providing an unprecedented opportunity to exhibit differences in TME across cancer types.^12^ Because different types of tumors have different TME characteristics, it is crucial to understand the internal and external factors leading to TME formation.^19^

The types of T cells and tissue stem cells in CRLM samples were found to be roughly the same. Colorectal cancer samples had a higher proportion of epithelial, NK, B, and NK T cells, whereas T cells were more prevalent in LM samples.^20^ The decrease of NK, NKT and other immune cells in LM samples may promote tumor immune escape to adapt to the liver microenvironment. Current studies have shown that the direction of differentiation of single cells influences changes in the tumor microenvironment, and that immunosuppressive TME is a major obstacle to immunotherapy.^19^ Monocytes differentiate preferentially into immunosuppressive tumor-associated macrophages (TAM) rather than immunostimulant dendritic cells (DC) in solid tumors.^21^ This study demonstrated that genetic suppression of retinoic acid (RA) production in tumor cells or drug suppression of RA-signaling in TME can increase irritant monocyte-derived cells, enhance T-cell-dependent anti-tumor immunity, and act synergistically with immune checkpoint suppression.

Different T cell subtypes play different roles in the tumor microenvironment, and exploring the T cell subtypes provides the research basis for improving the therapeutic effect.^19^ These subtypes include MAIT, VD2, GDT, T regulatory cells, and other cell types, among which MAIT and Treg cells occupy a certain proportion.^22^ The MAIT cells belong to another discrete atypical T cell subset characterized by a limited T cell receptor (TCR) library. As a kind of γδT, VD2Vδ2γδT lacks the expression of CD4 and CD8 and plays an important role in anti-tumor immunity with 0.5–16% abundance in circulation and lymph nodes.^23^ Not only do T regulatory cells inhibit the function of T cells, but further functional enrichment analysis found that T cells are mainly involved in the regulation of cytokine-cytokine receptor interaction pathway, antigen processing and presentation, and chemokine signaling pathway. Chemokine signaling pathway enrichment explains the hyperinfiltration of T cells in rectal LM. The depleted immune cells are inhibited in their ability to kill tumor cells, and the Treg cells further inhibit the anti-tumor immunity of T cells.

In cancer stem cell types, the differentially regulated genes in different samples are mainly involved in the interleukin (IL)-17-signaling pathway, the tumor necrosis factor (TNF)-signaling pathway, and the like. Analysis of the top five up- and downregulated genes in LM samples and primary colorectal cancer showed that most of the genes were closely related to prognosis. Targeted intervention of key genes in cancer stem cells is expected to inhibit tumor progression, and the development of new drugs is expected to bring hope to patients.

To explore the biologic process of CRLM at the cellular level, we used samples of primary CRC and corresponding LM to explore the regulatory functions of differentially expressed genes (DEGs) in different cell types. At the same time, we analyzed the protein-protein interaction network of these differentially expressed genes and found the mutual effects of different cell types in CRLM. Because T cells belong to the largest cell population, we re-clustered these cells and analyzed the biologic functions of the differentially expressed genes in the re-clustered cells.

To explore the differences between CRC and CRLM samples, we performed differential analysis and found the top five differentially up- and downregulated genes in LM samples. The TCGA database was used for expression analysis and survival curve analysis, and the findings showed that these genes were involved in the important process of CRC and liver cancer. They can potentially be used as a biomarker for research and provide theoretical support for further exploration of CRLM.

In addition, we found that SPARC was highly expressed in CRLM, and that its expression was positively correlated with LGR5, a marker of CRC stem cells. In further cell experiments, we confirmed that knockdown of SPARC could reduce the cell sphere-formation ability, the colony-formation ability, invasion, and the migration ability of CRC cells, and that the cell sphere-formation ability, the migration ability, the colony-formation ability, and invasion were enhanced after overexpression SPARC. We further found that SPARC expression was elevated in lymph node metastasis. These results suggested that SPARC may be the focus of future research.

Due to funding and time constraints, we preliminarily validated only the role of SPARC in CRLM. We did not further explore the molecular mechanism through which SPARC regulates the occurrence and development of CRCLM. In future studies, we will validate SPARC as a diagnostic and preventive marker in larger clinical samples and explore the mechanism of SPARC in CRLM by in vitro and in vivo experiments.

## Conclusion

This study identified several key genes in the process of CRLM. The findings showed that SPARC was significantly upregulated in CRLM samples and corresponded to CRC stem cells. Furthermore, SPARC might be a potential target in the therapy of CRLM.

## Supplementary Information

Below is the link to the electronic supplementary material.

Supplementary file1 (DOCX 14 kb)

## Data Availability

Data sourced from the Gene Expression Omnibus (GEO) and The Cancer Genome Atlas (TCGA) databases.
