# Supplementary material for: Identification and Verification of Key Genes in Colorectal Cancer Liver Metastases Through Analysis of Single-Cell Sequencing Data and TCGA Data
Source: Ann Surg Oncol. 2024 Oct 9;31(13):8664–79. doi: 10.1245/s10434-024-16194-9 (PMC11549235; doi:10.1245/s10434-024-16194-9)

**Table S1. Clinical information of the patients.**

|  | **Gender** | **Age** | **CEA** | **CA199** | **Location of primary lesion** | **Number of liver metastases** | **Preoperative TNM-staging** | **Postoperative TNM stage** |
| --- | --- | --- | --- | --- | --- | --- | --- | --- |
| 1 | Male | 67 | 19.16 | 27.58 | Rectum | 2 | cT4bN2M1 Ⅳ | cT4bN2M1 Ⅳ |
| 2 | Male | 77 | 108.1 | 907.9 | Hepatic flexure of (colon) | 4 | cTxNxM1 IV | yPT4aN1aM1a IVA |
| 3 | Male | 66 | 17.5 | 34.08 | Junction between rectum and sigmoid | 1 | IV | ypT4aN1bM1b IV |
| 4 | Male | 68 | 3.4 | 37.35 | Rectum | 1 | cT4aN+M1 IV | ypT3N1aM1a IVa |
| 5 | Male | 63 | 5.2 | 19.4 | Rectum | 1 | cT3N+M1a IV | ypT3N1cM1a IV |
| 6 | Male | 66 | 5.3 | 43.54 | Sigmoid flexure | 3 | IV | ypT3N1aM1 IV |
| 7 | Male | 58 | 4.8 | 6.33 | Sigmoid flexure | 5 | IV | ypT3N1bM1a IV |
| 8 | Male | 40 | 2.42 | 13.62 | Ascending colon | 9 | cT4aNxM1 IV | ypT0N0M1a IVA |
| 9 | Male | 36 | 3.86 | 9.94 | Hepatic flexure of (colon) | 3 | cT4aN+M1 IV | ypT4aN1cM1b IV |
| 10 | Female | 61 | 3.52 | 1.34 | Sigmoid flexure | 2 | cT4aNxM1 IV | cT4aNxM1 IV |
| 11 | Male | 58 | 2.39 | 2203 | Rectum | 1 | cT3bN+M1 IV | ypT3N0M1a IV |
| 12 | Male | 63 | 29.5 | 23.23 | Ascending colon | 5 | cT4aN2M1 IV | ypT3N0M1a IVA |
| 13 | Male | 52 | 42.3 | 96 | Sigmoid flexure | 1 | cT3N+M1a IV | ypT3N0M1a IVA |
| 14 | Female | 58 | 31.8 | 65 | Rectum | 1 | cT3N1M1 IV | ypT2N2aM1 IV |
| 15 | Female | 59 | 3.8 | 47 | Rectum | 1 | cT3NxM1 IV | ypT3N2aM1 IV |
| 16 | Male | 60 | 75.6 | 0.48 | Sigmoid flexure | 7 | cTxN0M1 IV | ypT3N1bM1a IVA |
| 17 | Male | 67 | 34.4 | 16.75 | Sigmoid flexure | 3 | cTxNxM1 IV | ypT0N0M1a IVA |
| 18 | Male | 73 | 1.8 | 9 | Sigmoid flexure | 3 | cT4NxM1 IV | ypT3N0M1 IV |

**Figure S1.** **Bubble plots of expression of the top 4 genes in different clusters.**

**Figure S2.** **Functional enrichment analysis of B cells.** A. Bubble map of B cells involved in major KEGG pathways. Larger bubbles indicated more genes. KEGG, Kyoto Encyclopedia of Genes and Genomes. B. Circle diagram of B cells involved in major GO-BP pathways. GO, Gene Ontology. BP, Biological Process.

**Figure S3.** **Functional enrichment analysis of T cells.** A. Bubble map of T cells involved in major KEGG pathways. Larger bubbles indicated more genes. KEGG, Kyoto Encyclopedia of Genes and Genomes. B. Circle diagram of T cells involved in major GO-BP pathways. GO, Gene Ontology. BP, Biological Process.

**Figure S4. Expression of DEGs in T cells, B cells and Monocyte.** A. Violin plot of DEGs expression in T cells. B. Violin plot of DEGs expression in B cells. C. Violin plot of DEGs expression in Monocyte. DEGs, differentially expressed genes.

**Figure S5.** **Expression of DEGs in NK cells, CRC cells and tissue stem cells.** A. Violin plot of DEGs expression in NK cells. DEGs, differentially expressed genes. NK, natural killer. B. Violin plot of DEGs expression in CRC cells. CRC, colorectal cancer. C. Violin plot of DEGs expression in tissue stem cells. DEGs, differentially expressed genes.

**Figure S6. Bar plot of CRC cell GO enrichment. CRC, colorectal cancer. GO, Gene Ontology.**

**Figure S7.** **The expression level of SPARC was increased in patients with lymph node metastasis.** A. The expression of SPARC in TCGA-COADREAD in the primary tumors (n = 207) and metastatic lesions (n = 168). ^**^ *p*<0.01. B. The expression of SPARC in GSE39582 in the primary tumors (n = 314) and metastatic lesions (n = 243). ^*^ *p*<0.05. C. The expression of SPARC in GSE29621 in the primary tumors (n = 314) and metastatic lesions (n = 243). ^*^ *p*<0.05.


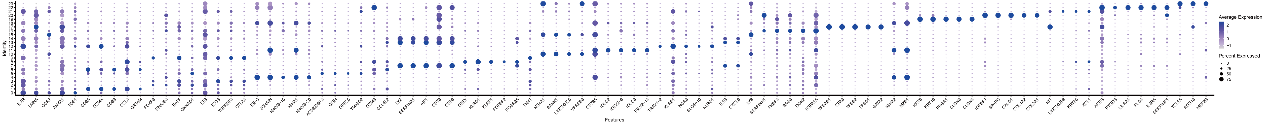


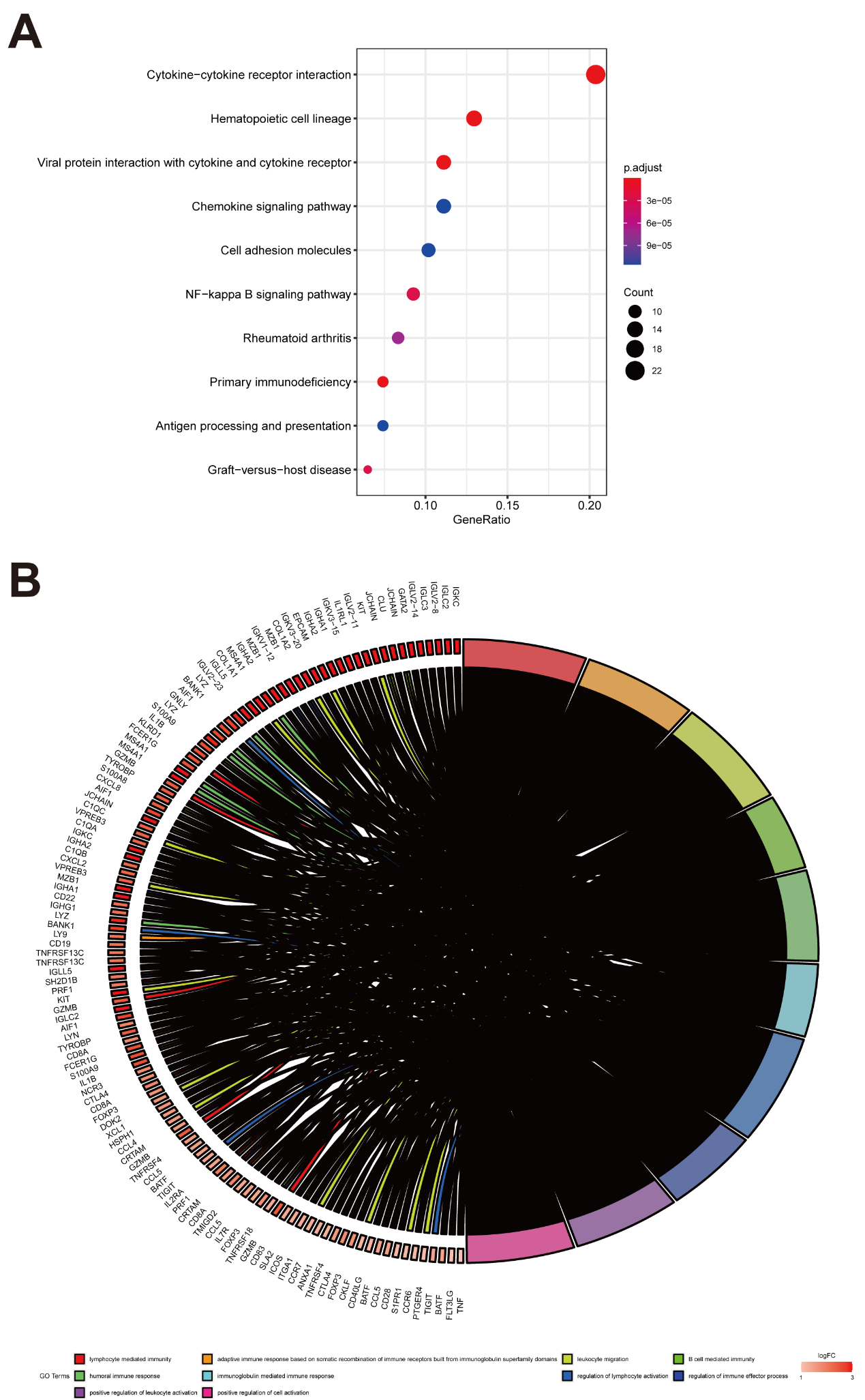


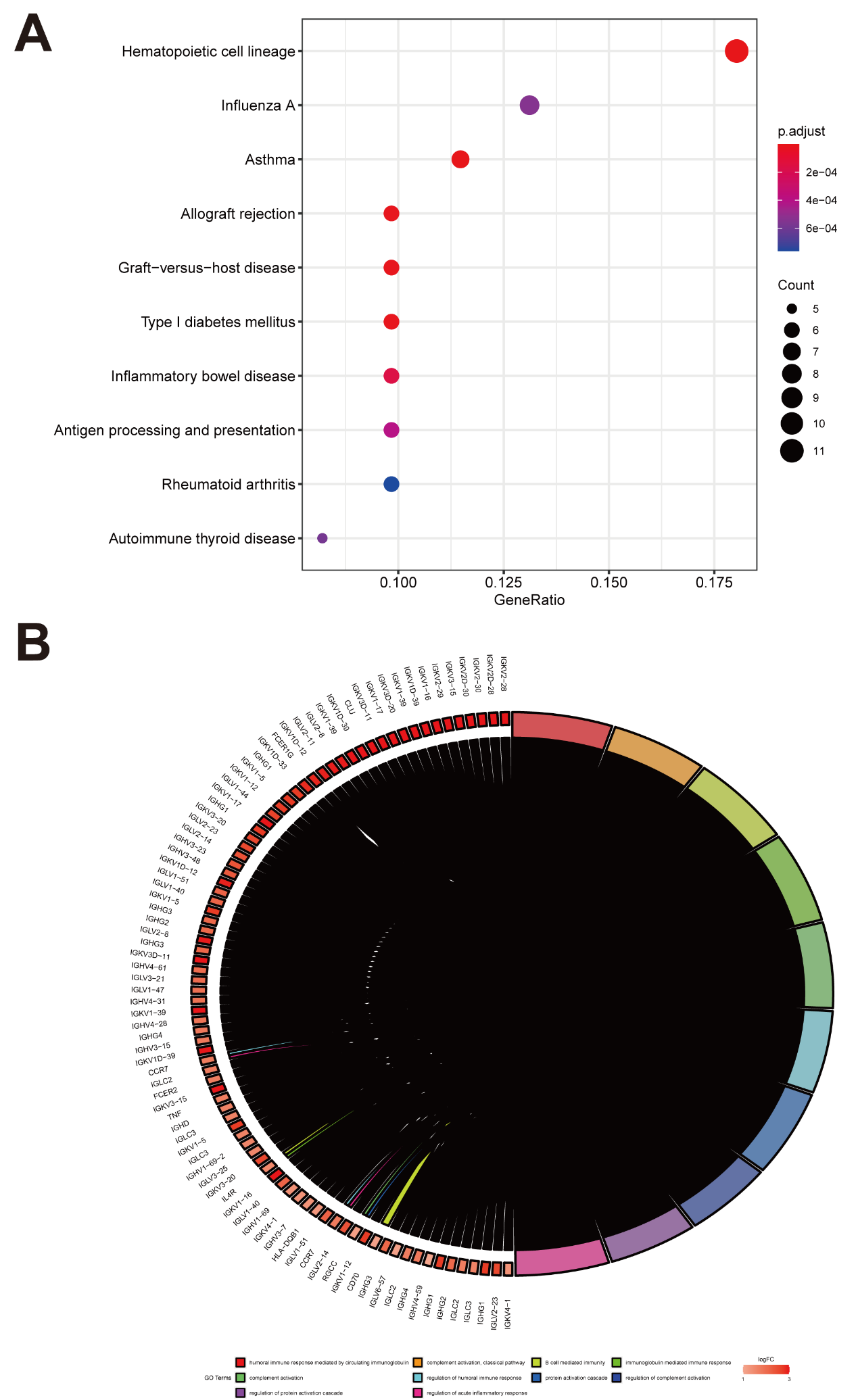


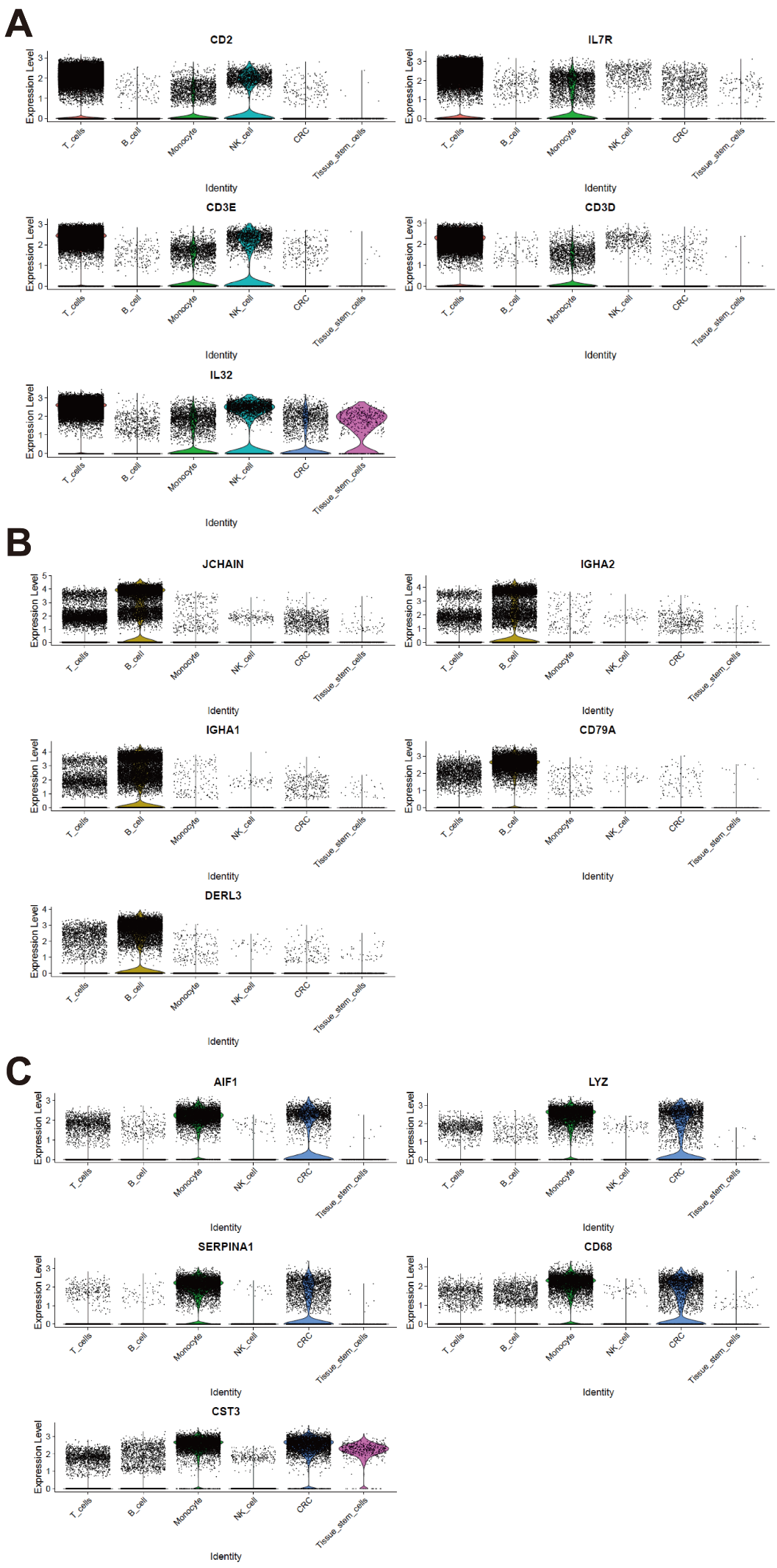


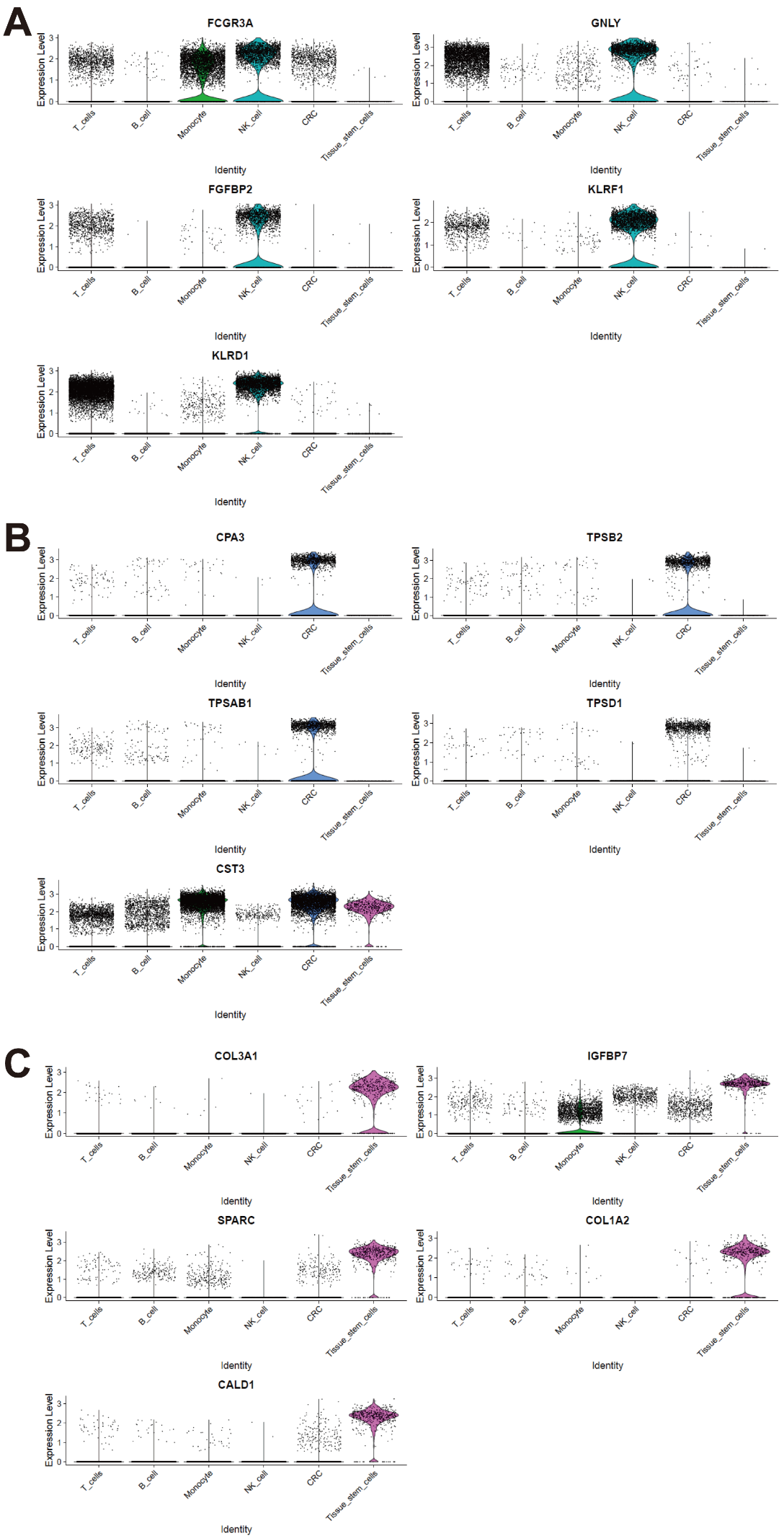


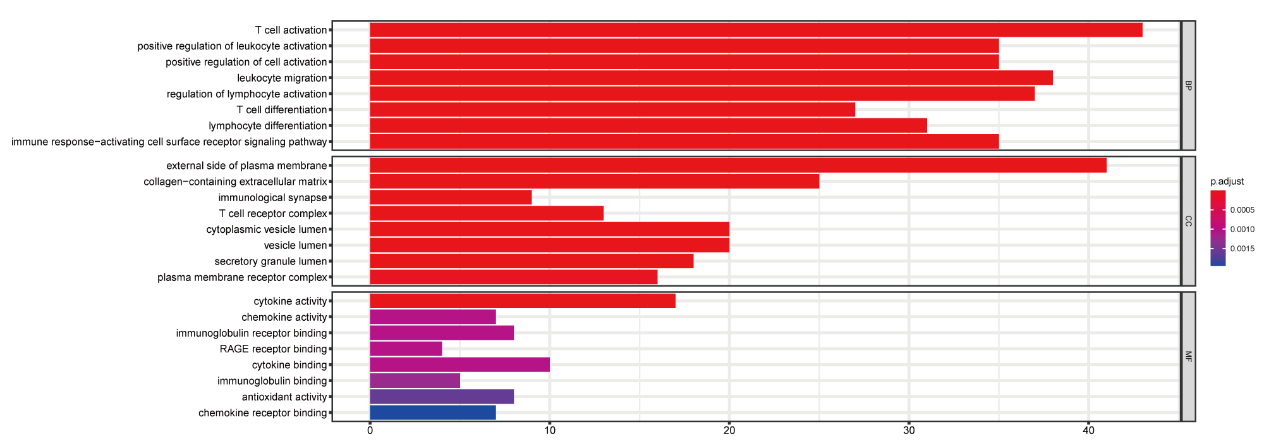


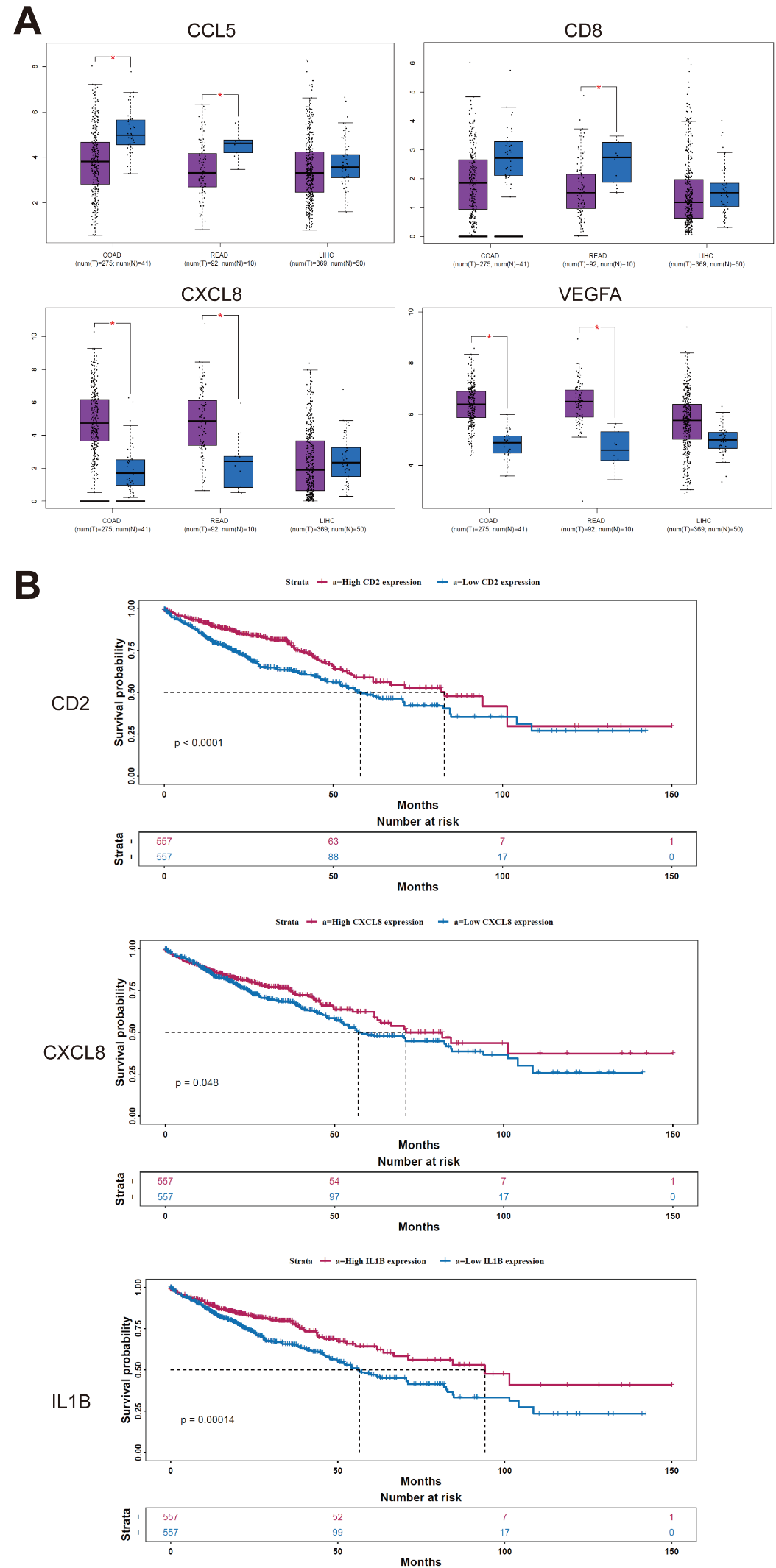

Supplement: Supplementary file 1 — Supplementary file1 (DOCX 14 kb) [file 10434_2024_16194_MOESM1_ESM.docx]
